# Can we reduce the stigmatisation experience with psychosocial interventions? An investigation of the meeting centre support programme impact on people with cognitive impairments

**DOI:** 10.1192/j.eurpsy.2021.378

**Published:** 2021-08-13

**Authors:** K. Lion, D. Szcześniak, S. Evans, S. Evans, E. Farina, D. Brooker, R. Chattat, F. Meiland, R.-M. Droes, J. Rymaszewska

**Affiliations:** 1 Menzies Health Institute Queensland, Griffith University, Nathan, Australia; 2 Department Of Psychiatry, Wroclaw Medical University, Wroclaw, Poland; 3 Association For Dementia Studies, University of Worcester, Worcester, United Kingdom; 4 Fondazione Don Carlo Gnocchi, IRCCS, Milan, Italy; 5 Department Of Psychology, University of Bologna, Bologna, Italy; 6 Department Of Medicine For Older People, Amsterdam UMC University Medical Centres, Amsterdam, Netherlands; 7 Amsterdam Public Health Research Institute Department Of Psychiatry, Amsterdam UMC, Amsterdam, Netherlands

**Keywords:** dementia, Stigma, attitude, social isolation

## Abstract

**Introduction:**

People living with dementia or mild cognitive impairment (MCI) experience stigmatisation and there are not many specific psychosocial interventions dedicated to help them coping with this issue, reducing its impact on their lives.

**Objectives:**

This study aimed to a) investigate the stigmatisation level among people with dementia and MCI in Poland, Italy and the United Kingdom and b) assess the role of the Meeting Centre Support Programme (MCSP) in decreasing stigmatisation.

**Methods:**

We investigated outcomes for 114 people with dementia and MCI living in Italy, Poland and the UK who participated 6 months in MCSP or usual care (UC) using a pre/post-test control group study design. Level of stigmatisation was assessed with the Stigma Impact Scale: neurological impairment (SIS).

**Results:**

Stigmatisation level (SIS) among participants varied from 2 to 65 (median=33.5; Q1=27; Q3=41) with people from the UK experiencing a statistically significantly higher level of stigmatisation than people in Italy and Poland. In Italy, stigmatisation was lower (p=0.02) in the MCSP group following the intervention. In Poland, the social isolation level did not significantly change in MCSP, but increased (p=0.05) in UC. In the UK, the social rejection level raised (p=0.03) in MCSP. Overall, the combined data of the three countries did not show statistically significant differences in SIS between MCSP and UC.

**Conclusions:**

Stigmatisation among people with dementia and MCI is complex and seems culturally dependent. There is a great opportunity in psychosocial interventions to reduce the burden of stigma among people with dementia which requires further investigation.

**Disclosure:**

No significant relationships.

